# Symptom Network Analysis Tools for Applied Researchers With Cross-Sectional and Panel Data – A Brief Overview and Multiverse Analysis

**DOI:** 10.1177/00332941231213649

**Published:** 2023-11-09

**Authors:** René Freichel

**Affiliations:** Department of Psychology, 1234 University of Amsterdam, Amsterdam, The Netherlands; Department of Psychology, Harvard University, Cambridge, MA, USA

**Keywords:** network analysis, psychopathology, panel data models, multiverse analysis

## Abstract

In recent years, there has been a growing interest in utilizing symptom-network models to study psychopathology and relevant risk factors, such as cognitive and physical health. Various methodological approaches can be employed by researchers analyzing cross-sectional and panel data (i.e., several time points over an extended period). This paper provides an overview of some commonly used analytical tools, including moderated network models, network comparison tests, cross-lagged network analysis, and panel graphical vector-autoregression (VAR) models. Using an easily accessible dataset (easySHARE), this study demonstrates the use of different analytical approaches when investigating (a) the association between mental health and cognitive functioning, and (b) the role of chronic disease in mediating or moderating this association. This multiverse analysis showcases both converging and diverging evidence from different analytical avenues. These findings underscore the importance of multiverse investigations to increase transparency and communicate the extent to which conclusions depend on analytical choices.

In recent years, symptom network modeling has emerged as a promising approach in the field of psychopathology. Most published network analysis studies are based on cross-sectional data or panel studies in which a group of individuals have been assessed at multiple time points (waves) over an extended period. This increase in popularity of applications of symptom network approaches has inspired the development of various methodological approaches that applied researchers can use. The goal of the present study is to provide an overview of important analysis tools and showcase their parallel use when investigating a substantial research question in the field of clinical psychology.

The initial notion that psychopathology emerges from the dynamic, causal interactions between symptoms motivated the development of a network theory of psychopathology (Borsboom & Cramer, 2013; Cramer et al., 2010). These theoretical considerations have spurred countless studies that have characterized the symptom network structure of common mental disorders, such as depression (e.g., Belvederi Murri et al., 2020), anxiety (e.g., McElroy et al., 2018), and addiction (Freichel et al., 2023a). Moreover, network analysis has allowed researchers to pinpoint symptoms that are highly interconnected and thus, they may carry a high relative importance (strength centrality) in the network (Elliott et al., 2020). However, the interpretation of centrality measures remains a point of debate, as several studies have raised concerns regarding psychometric theory and underlying assumptions of these centrality indices (Bringmann et al., 2019; Hallquist et al., 2021).

A network is essentially a graphical representation consisting of both nodes (variables) and edges (associations between variables). As such, the versatile use of network modeling approaches, as a statistical toolbox, has expanded beyond the focus of symptoms to also integrate other sources of data in a network, including brain markers (Blanken et al., 2021; Freichel et al., 2023b), polygenic risk scores (Isvoranu et al., 2020) proteins and lipid markers (Zainal & Newman, 2022), as well as performance-based measures of cognition (Zainal & Newman, 2023). Thus, network modeling has become a useful tool for investigating complex relationships among various factors in a range of different domains.

The progress in the field of network psychometrics has led to the development of a multitude of analytical approaches (see Figure 1), including *network comparison tests* (van Borkulo et al., 2022) that allow for the comparisons of network structures, as well as, *moderated network models* (Haslbeck et al., 2021a) that allow researchers to test moderation effects inside a network. For instance, researchers may have explicit hypotheses regarding the impact of age on the connections (edges) between different depression symptoms within a network. Moderated network models provide a useful tool for examining whether these connections change as age increases.

**Figure 1.**
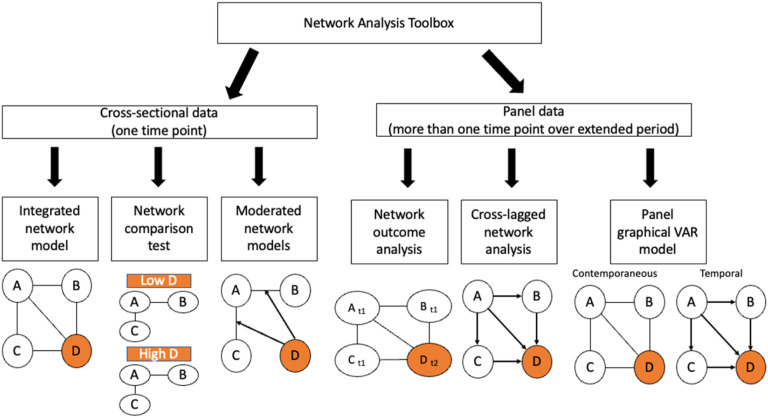
Overview of important network analysis tools for cross-sectional and panel data. *Note.* This overview displays frequently employed analytical approaches for cross-sectional (i.e., one time point) and panel (i.e., several time points across an extended period) data. The variable (node) representing the construct of interest is highlighted in orange (grey). Integrated network models include the construct of interest as a mediating node; moderated network models include the construct of interest as a moderator. Network outcome analysis integrates prospectively assessed outcomes (subscript t2) and other baseline variables (subscript t1).

In addition to the analysis of cross-sectional data, there has been a growing focus on analyzing intensive time-series (through ecological momentary assessment) or panel data in recent times. Longitudinal (network) analysis approaches allow researchers to study (within-person) temporal dynamics, both over shorter time-scales, such as daily windows (e.g., see Ebrahimi et al., 2021) as well as longer time scales, such as years during childhood (e.g., see Speyer et al., 2021). Analytical approaches, such as *panel graphical vector-autoregression (GVAR) models* (Epskamp, 2020) and *cross-lagged panel network analysis (CLPN)* models (Rhemtulla et al., 2022) have been developed as tools for longitudinal network analysis. In addition, network outcome analysis (Blanken et al., 2020) has been proposed as a tool to examine predictive associations between nodes assessed at single waves (*t*) and outcomes assessed at future waves (*t+1*).

The present study aims to provide a brief overview of different methodological approaches in the field of network psychometrics for the analysis of cross-sectional and panel data. Using an easily accessible dataset and open analysis scripts, it aims to illustrate how different analytical approaches can be used to investigate the same substantial research question.

## Illustrative Research Question

To illustrate the use of different network analytical approaches, the focus of this report revolves around the following research questions: (1) What is the relationship between mental health/well-being and cognitive functioning? (2) Does physical health play a role in mediating or moderating this association? Drawing from an extensive body of existing literature and a recent study using the Survey of Health, Ageing and Retirement in Europe (SHARE) data (Csajbók et al., 2022), we predicted that physical health (indicated through number of chronic diseases) may mediate the association between cognitive functioning and mental health/well-being.

## Data Source

We used data from the SHARE study (Börsch-Supan et al., 2013). This international longitudinal panel study was collected at multiple sites in Europe and Israel with the goal to examine the effects of social, health, and economic policies. All participants provided informed consent and the project received ethical approval by the Ethics Council of the Max Planck Society. The data is openly accessible after registration on their website (www.share-project.org). For the tutorial purpose of the present study, we used the easySHARE dataset. This simplified dataset contains the same number of observations as the main release; however, it is limited to a set of important variables.

The SHARE study included individuals aged 50 years or older with a residence in any of the SHARE countries. We included data from four different waves (waves 1, 2, 4, 5) as all measures of interest have been assessed during these waves of data collection. The different waves were spaced apart by two years, except for wave 4 which was four years after wave 3. For all cross-sectional analyses, we used the data from the first wave of data collection. The first wave included 17,945 participants who provided valid information for all measures of interest. The sample is mostly balanced with respect to gender (55.05% identified as female), with most individuals in the early retirement stage (mean age = 63.66, *SD* = 10.11), and 72.47% are married and living together with their spouse. Table S1 in the supplementary materials provides more details on the sociodemographic sample characteristics.

We included three different measures of mental health/well-being, including depression symptoms, frequency of drinking alcohol, and quality of life. Two cognitive tasks assessed cognitive functioning, specifically, memory and mathematical performance. As an indicator of physical health, we included individuals’ self-reported number of chronic diseases. A detailed description of all measures is provided in the supplementary materials section 2. All analyses were conducted in RStudio, and we provide all analysis scripts, including data inspection, preprocessing, and model estimation scripts on the Open Science Framework (OSF, https://osf.io/vqzsy/?view_only=fe79b6f659b648d697389e9109ff7962).

## Cross-Sectional Network Approaches

### Integrated Cross-Sectional Network Model

One commonly used approach to study the association between different constructs of interests is through a cross-sectional (e.g., partial correlation) network model. Several estimation procedures are available for different data distributions, including Gaussian graphical models (GGM) for multivariate normally distributed data (Epskamp et al., 2018), Ising models for binary data (van Borkulo et al., 2014), and mixed graphical models (Haslbeck & Waldorp, 2015) for mixed data (continuous, count, categorical). Recently, Bayesian network estimation procedures have been developed that offer information about the network structure uncertainty and evidence for edge inclusion and exclusion (see Huth et al., 2023 for a tutorial and overview). To identify causal relations in cross-sectional networks, directed acyclic graphs (DAGs) have been proposed. These probabilistic graphical models visualize conditionally independent associations between variables and they assume (1) the presence of all important causal variables (*sufficiency)*, and (2) the presence of probabilistic dependence or only directed edges between nodes (see Briganti et al., 2022 for a tutorial on DAGs).

To illustrate the utility of integrated network models, we have estimated a cross-sectional network (GGM) that includes measures of mental health, cognitive functioning, and the mediating construct of interest (number of chronic diseases) as nodes in the network. The cross-sectional network was estimated using the *bootnet* R package (Epskamp & Fried, 2015) and visualized using the *qgraph* R package (Epskamp et al., 2012). We used the “EBICglasso” algorithm that implements the least absolute shrinkage and selection operator (LASSO) regularization method. Considering the large number of associations and potential overfit, this regularization penalizes coefficients and results in a sparser network. We selected a standard tuning parameter of .5 that shrinks weak coefficients to zero. A non-parametric bootstrapping procedure was used to examine the stability of the estimated edge weights (see Figure S1).

**Figure 2.**
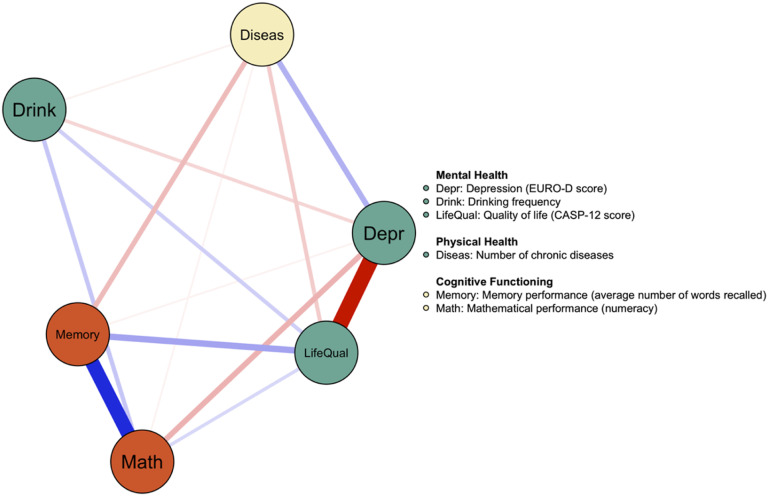
Integrated Cross-sectional Network at Wave 1. *Note.* The strength of the associations is represented in the thickness and color saturation of the edges. Positive associations are displayed in blue; negative associations are shown in red. The color of the nodes represents the domain that the variables belong to.

Figure 2 displays the resulting network model that shows particularly strong associations between nodes within the same domain (depressive symptoms-life satisfaction; memory – mathematical performance). We found that a higher number of chronic diseases was associated with more depressive symptoms, lower quality of life, and lower memory performance. In this context, physical health (number of chronic diseases) is conceptualized as a mediating factor.

### Moderated Network Models

The integrated cross-sectional network conceptualizes chronic diseases as a mediating node that shows pairwise associations with other variables. An alternative approach to studying the role of physical health is through moderated network models. These moderated network models allow us to test whether the construct of interest (e.g., number of chronic diseases) moderates the pairwise associations between existing nodes in the network. For instance, we may predict that the association between depression symptoms and cognitive functioning is particularly strong in individuals with poor physical health as access to healthcare resources may be more limited.

Moderated network models can be estimated in a nodewise regression approach using the *mgm* R package (Haslbeck & Waldorp, 2020). A moderation effect represents a three-way interaction effect between the moderator and the pairwise association between two nodes. To better understand and visualize moderation effects, one can condition the network on a scaled value of the moderator. A full description of the method and tutorial can be found elsewhere (Haslbeck, 2022).

**Figure 3.**
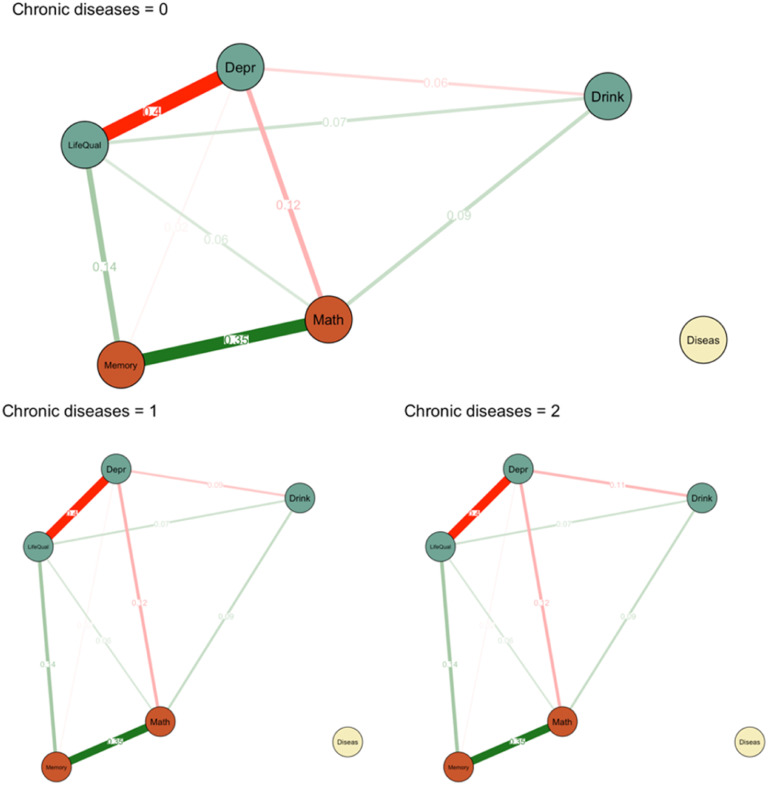
Conditioned networks. *Note.* There is a significant moderation effect of number of physical diseases on the association between depressive symptoms (‘Depr’) and drinking frequency (‘Drink’). Positive associations are displayed in green; negative associations are shown in red.

We found a weak but significant moderation effect (interaction weight: −.024) of chronic diseases (moderator) on the association between depression symptoms and drinking frequency. This suggests that the pairwise association between depressive symptoms and drinking frequency becomes weaker as the number of chronic diseases is increasing. Figure 3 shows the networks conditioned in different levels of chronic disease. A bootstrapping procedure was used to obtain stability estimates for the moderation effects (see Figure S2 in the supplementary materials).

### Network Comparison Tests

Instead of modeling continuous moderation effects, researchers commonly use a split-sample approach to examine moderation effects of a variable of interest. In this approach, the sample is split according to the measure of interest (moderator) and two separate networks (excluding the moderator) are estimated and compared using network comparison tests. These permutation-based hypothesis tests can be used to assess differences in each edge parameter between two networks. The R package *NetworkComparisonTest* (van Borkulo et al., 2022) can be used to conduct these tests. A recent study describes the limitations of this split-based approach and uses simulations to showcase that moderated network models may prove more adequate for testing moderation effects (Haslbeck et al., 2021a).

**Figure 4.**
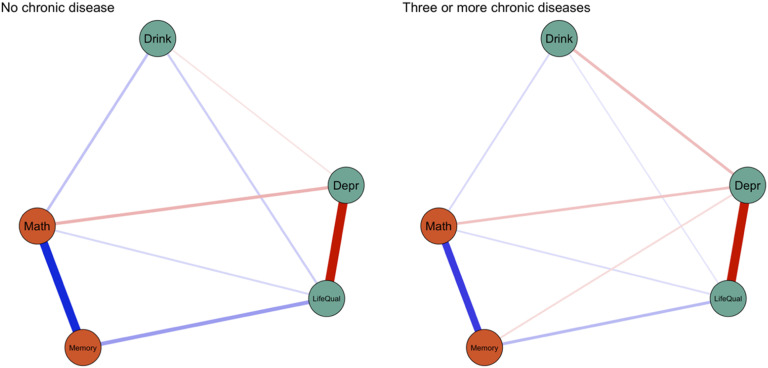
Split-sample networks at wave 1.

To illustrate the use of network comparison tests, we first split the sample into two extreme groups (Group 1: no chronic diseases, Group 2: three or more chronic diseases), estimated separate networks (see Figure 4), and used network comparison tests to test for edge differences. We predicted that in the chronic disease group, there would be stronger associations between cognitive functioning and mental health/well-being.

The network comparison test have been estimated using 1000 bootstraps and the Holm method for multiple comparison correction (Holm, 1979). The networks differed in structure (*M* = .13, *p* < .05) and Table 1 lists the significantly different edges between the groups. The network in the disease group shows significantly weaker associations between depression and drinking frequency, life satisfaction, and memory performance. This significant edge difference found between depression symptoms and drinking frequency is consistent with the moderation effect described above.

**Table 1.** Significant Group Differences Based on Network Comparison Test (After Holm Correction).

Edge Comparison	No Diseases Network	Three or More Chronic Diseases Network
Depression – Drinking frequency	−.04	−.13
Depression – Life satisfaction	−.36	−.50
Depression - Memory	.00	−.07

*Note.* The table includes the significantly different edge weights in the two groups.

The different approaches presented above (integrated network, moderated network models, network comparison tests of split groups) represent three commonly used methods in the field of network psychometrics. There are various other approaches that can be used to answer similar questions. For instance, a *network tree* approach (Jones et al., 2020) uses model-based recursive partitioning to explain heterogeneity in the network structure. After recursively splitting the sample based on certain split variables, a GGM is estimated in the subgroup, and fit statistics are computed based on log-likelihood. This tree-based approach determines whether different predictors affect the fit statistic, and it results in a split with the highest fit improvement. This exploratory tool may be useful in cases where researchers aim to study a range of different context variables and determine their relative contribution to heterogeneity in the network structure. For instance, one may compare the role of chronic diseases with other relevant factors associated with physical health, including physical activity.

## Longitudinal Network Approaches

To understand how different factors predict each other over time, longitudinal network approaches provide valuable tools. Three different approaches (network outcome analysis, cross-lagged network analysis, and panel graphical vector autoregressive models) will be discussed. These tools allow researchers to understand the directionality of associations and they may offer insights into potential causal relationships.

### Network Outcome Analysis

Network outcome analysis (see Blanken et al., 2020) represents a special case of an integrated network model in which different predictors (at wave t1) and outcomes (at wave t2) are integrated into one network. This approach allows researchers to examine whether there are any direct predictive associations between predictors and outcomes after controlling for all other associations between variables at t1. This approach is particularly useful in situations in which important (outcome) variables are only available at certain waves of data collection.

### Cross-Lagged Network Analysis

**Figure 5.**
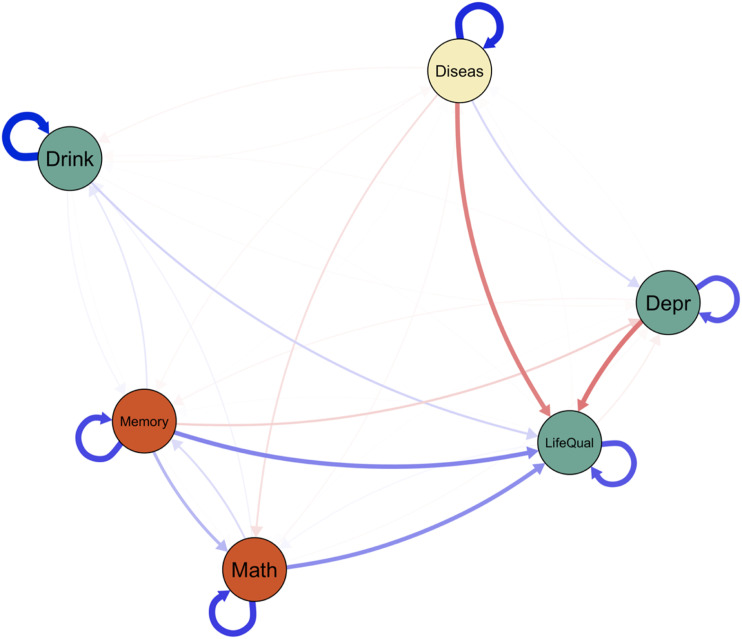
Temporal network based on CLPN analysis.

To examine the associations between different variables over time, cross-lagged panel network (CLPN) analysis (Wysocki et al., 2022) can be used. Using the *glmnet* R package (Friedman et al., 2017), regularized regressions estimate cross-lagged associations between all nodes after accounting for their autoregressive effects (i.e., predicting itself). The estimation procedure includes LASSO regularization to penalize estimates and 10-fold cross-validation for the tuning parameter. The directed temporal lag-1 associations displayed in the network (see Figure 5) visualize the weights of the regression estimates. These temporal associations represent directed effects after adjusting for all other variables at the first wave (Schlechter et al., 2022). We assumed data to be missing at random and used complete case analysis. We found that the number of chronic diseases showed strong autocorrelations and it predicted lower quality of life, more depression symptoms, and lower cognitive (mathematical) performance over time (see Figure 5).

This CLPN approach is particularly useful when studying single changepoints (wave 1 to wave (2) as other longitudinal network approaches, such as panel graphical vector-autoregression (GVAR) models (Epskamp, 2020) require at least three waves of data. An important limitation of the CLPN approach (and other cross-sectional network approaches) concerns their inability to separate within- and between-person variance. This is crucial as insights about mechanistic pathways that form the basis of many theories in (developmental) psychopathology require a disaggregation of within-person and between-person effects (Curran & Bauer, 2011).

### Panel Graphical Vector-Autoregression Models

To separate within- and between-person effects, panel GVAR models (Epskamp et al., 2018) can be used. These GVAR models are structurally similar to the random-intercept cross-lagged panel model (Hamaker et al., 2015) and they require at least three waves of data. Every variable is predicted by itself and the cross-lagged values of all other variables in the VAR part of the model which results in autoregressive and temporal cross-lagged estimates. The innovation is modeled in a GGM to obtain contemporaneous associations of different variables within the same window. A key feature of this model is the separation of average within-person temporal (effects over time) and contemporaneous (associations within the same time window) effects. First, a saturated model which includes all edges is fitted to the data. Second, standard pruning procedures (see Blanken et al., 2022) can be used to remove non-significant associations. The model fit is evaluated according to widely used criteria in the structural equation modeling field (Kline, 2005; Sivo et al., 2006). The Root Mean Square Error of Approximation (RMSEA), Comparative Fit Index (CFI), and the Tucker-Lewis index (TLI) are standard indices for good model fit (RMSEA < .05, CFI > .95, TLI > .95). Panel GVAR models can be estimated using the *psychonetrics* (Epskamp, 2021) R package. Recently, the panel GVAR model has been employed to investigate dynamic changes during socioemotional development (Speyer et al., 2022) and adolescent substance use (Freichel et al., 2023c).

To illustrate, we have fitted a panel GVAR model to four waves of data (waves 1, 2, 4, 5). The third measurement wave did not contain all relevant measures of interest (e.g., depression symptoms, drinking frequency) so we included an empty third wave of data to adhere to the statistical assumption of equidistant measures. This served as a data analytical technique to ensure the estimation of a consistent time lag between waves in the model. Full-Information-Maximum-Likelihood (FIML)  estimation was used to account for missing data. We have removed linear and quadratic trends to facilitate stationarity. This is common in applications of graphical VAR models that explicitly focus on the correlational, and not mean structure of the data (Freichel et al., 2022; Speyer et al., 2021). The saturated model showed an excellent fit to the data (TLI = .98, CFI = .99, RMSEA = .013, BIC = 2401595.12). Standard pruning procedures (with alpha = .05) were applied to make the model robust to false positives. The pruned model (TLI = .99, CFI = .99, RMSEA = .012, BIC = 2401385.31) is more interpretable considering its sparse network structure.

**Figure 6.**
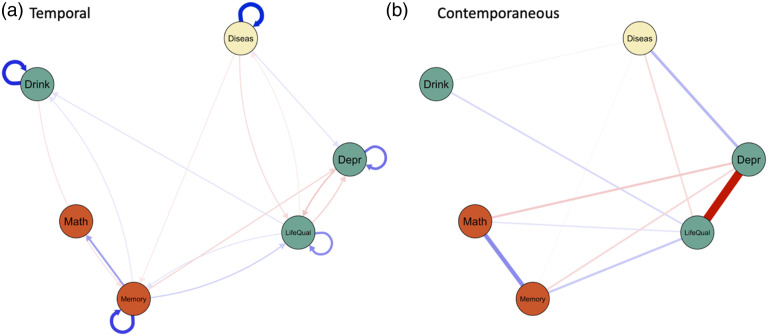
Within-person temporal and contemporaneous networks based on panel GVAR model. *Note.* The temporal associations (panel A) represent directed partial correlations.

Figure 6 shows the within-person temporal (panel A) and contemporaneous (panel B) associations. Physical health (number of chronic diseases) predicted more depression symptoms and lower memory performance over time. We also found a negative association between quality of life and physical health. A higher quality of life predicted fewer chronic diseases. The edge in the reverse direction (chronic diseases predicting lower life quality) was considered unstable when inspecting the bootstrapping results (see Figure S3). Within the same time window, chronic disease co-occurs with more depression symptoms, lower quality of life, more frequent drinking, and lower memory performance.

**Figure 7.**
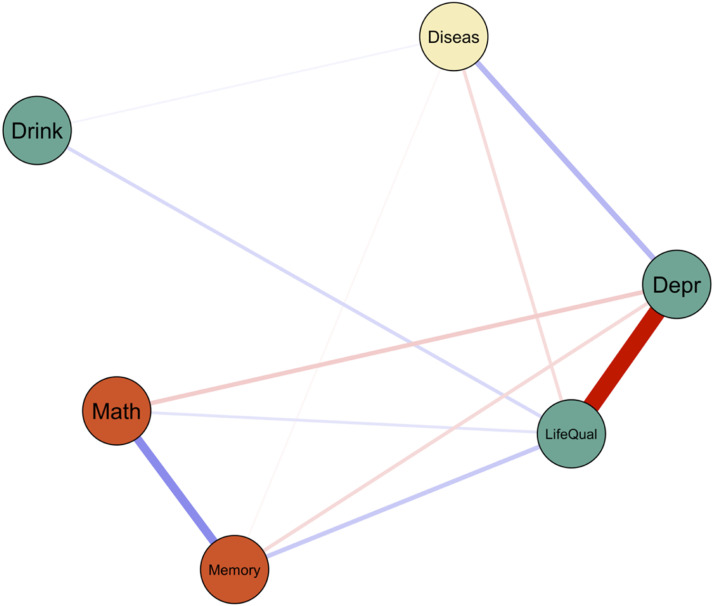
Between-person network.

The between-person network describes the interrelationship between means of subjects, representing trait-like, time-invariant effects. As shown in Figure 7, different indicators of mental health, physical health, and cognitive functioning covary substantially at the trait level. For instance, participants with more chronic diseases also tended to report more depression symptoms and lower quality of life. Higher quality of life was associated with better memory and math performance.

To estimate the stability of the extracted network structures, a case-dropping bootstrapping analysis can be used. The final network model is estimated many times (e.g., 1000) with only 75% of the sample. The proportion of times an edge is present in each of the bootstrapped samples allows researchers to judge the stability of the network. Previously, edges included in more than 50% of times were considered stable (Zainal & Newman, 2022). The bootstrapping analysis indicated sufficient stability of the network structures (see Figures S3–S5).

There are several important methodological challenges. Panel GVAR models require at least three (equidistant) waves of data, and they can only capture linear dynamics. Moreover, considering the amount of data required, they are often used to assess lag-1 relationships. Likely, complex psychopathological processes operate on different time lags and show non-linear trends. Moreover, the panel GVAR model assumes stationarity, thus, making it difficult, to uncover gradual changes in development, particularly in situations, in which non-stationarity in means or covariances is expected. Thus, it is important to first describe and inspect the trends (across time) in the data before estimating panel GVAR models.

## Concluding Comments

This multiverse analysis showed both converging and diverging evidence from different analytical avenues. For instance, moderated network models identified a weak moderating effect of the number of chronic diseases on the association between depression symptoms and frequency of drinking. When using network comparison tests with extreme split-samples, this edge was significantly different which is consistent with the moderated network approach. However, two additional edges (depression symptoms–life satisfaction, depression symptoms–memory performance) also showed significant group differences in the network comparison tests. When studying temporal associations, we found even stronger differences between CLPN analysis and panel graphical VAR models. This may likely stem from the different model specifications that either conflate (CLPN) or separate (panel GVAR) within- and between-person effects.

The present study provided an overview of some commonly used network approaches for cross-sectional and panel data. These two sources of data remain among the most commonly used by applied researchers in clinical psychological science. It goes beyond the current overview to discuss group-level network estimation from time-series data (see Burger et al., 2022 for an overview), personalized network models (Mansueto et al., 2022), or other advanced approaches, such as time-varying network models (see Haslbeck et al., 2021b for a tutorial).

When applying these different tools, it is important to follow best practices and document all analytical decisions made in the process to increase reproducibility. Reporting standards for psychological network analyses can be found elsewhere (see Burger et al., 2023). Detailed information about the variable selection, model selection algorithms (e.g., pruning, thresholding), use of statistical software, visualization parameters (e.g., removing edges below certain values), proportion of missing data as well as a description of the network stability analysis (e.g., parametric/non-parametric bootstrapping; number of bootstraps) should be reported.

In addition to the methodological limitations of certain tools discussed above, there are several practical challenges that researchers may face when estimating psychological networks: (1) Psychological networks identify patterns of covariance, and thus when interpreting edges, it is important to consider nodes that violate distributional assumptions, lack sufficient variability, or show a large degree of multicollinearity. Thus, researchers should inspect their data and carefully consider which nodes to include in their model; (2) Considering the large number of parameters being estimated in psychological networks, it is crucial to conduct network stability analyses and reflect on the stability of specific edges (i.e., confidence intervals) when interpreting results, especially with smaller sample sizes; (3) When estimating networks in different subgroups, it is important to discuss the extent to which the groups are based on the variables included in the network. Simulation studies have shown that such scenarios may induce negative edges in the network as a result of Berkson’s bias (Ron et al., 2021). Thus, identifying groups based on latent class mixture models or independent criteria (i.e., measures not directly included in the network) may mitigate this risk.

Moreover, recently there has been increasing interest in confirmatory network analysis. Using the *psychonetrics* software (Epskamp, 2021), researchers can specify edge matrices with predicted pathways and fixed estimates that can be tested in existing datasets. This new tool may be particularly useful for researchers when testing developmental/process theories in psychopathology. Lastly, applied researchers should carefully review which analytical approach is most appropriate for their present research question and dataset. For cross-sectional analyses, determining whether a construct of interest should be included as a node in the network (mediation framework) or included as a moderator (moderated network models) largely depends on the theoretical framework. For longitudinal network approaches, the choice of analysis depends on the goals of the respective theoretical framework (e.g., testing within-person processes or prediction) and practical considerations with respect to the availability of data (e.g., at least three waves of data required for panel GVAR models). The set of approaches presented here, along with their accompanying analysis scripts, may serve as inspiration for applied researchers. Using several analytical approaches in parallel, as a multiverse investigation (Steegen et al., 2016) may increase transparency and communicate the extent to which conclusions depend on analytical choices.

## Supplemental Material

Supplemental Material - Symptom Network Analysis Tools for Applied Researchers With Cross-Sectional and Panel Data – A Brief Overview and Multiverse Analysis Supplemental Material for Symptom Network Analysis Tools for Applied Researchers With Cross-Sectional and Panel Data – A Brief Overview and Multiverse Analysis by René Freichel in Psychological Reports.

## Data Availability

The data given in this article are accessible after registration on the SHARE project website (www.share-project.org). The citation for the dataset is: Börsch-Supan, A. & S. Gruber (2020): easySHARE. Release version: 8.0.0. SHARE-ERIC. Dataset. doi: 10.6103/SHARE.easy.800 and Bergmann, M., T. Kneip, G. De Luca, and A. Scherpenzeel (2019). Survey participation in the Survey of Health, Ageing and Retirement in Europe (SHARE), Wave 1-7. Based on Release 7.0.0. SHARE Working Paper Series 41-2019. Munich: MEA, Max Planck Institute for Social Law and Social Policy. This paper uses data from the generated easySHARE data set (DOI: 10.6103/SHARE.easy.800), see Gruber et al. (2014) for methodological details. The easySHARE release 8.0.0 is based on SHARE Waves 1, 2, 3 (SHARELIFE), 4, 5, 6, 7 and 8 (DOIs: 10.6103/SHARE.w1.800, 10.6103/SHARE.w2.800, 10.6103/SHARE.w3.800, 10.6103/SHARE.w4.800,10.6103/SHARE.w5.800, 10.6103/SHARE.w6.800, 10.6103/SHARE.w7.800, 10.6103/SHARE.w8.800). This paper uses data from SHARE Waves 1, 2, 4, 5  (DOIs:  10.6103/SHARE.w1.800, 10.6103/SHARE.w2.800, 10.6103/SHARE.w4.800, 10.6103/SHARE.w5.800) see Börsch-Supan et al. (2013) for methodological details. The SHARE data collection has been funded by the European Commission, DG RTD through FP5 (QLK6-CT-2001-00360), FP6 (SHARE-I3: RII-CT-2006-062193, COMPARE: CIT5-CT-2005-028857, SHARELIFE: CIT4-CT-2006-028812), FP7 (SHARE-PREP: GA N°211909, SHARE-LEAP: GA N°227822, SHARE M4: GA N°261982, DASISH: GA N°283646) and Horizon 2020 (SHARE-DEV3: GA N°676536, SHARE-COHESION: GA N°870628, SERISS: GA N°654221, SSHOC: GA N°823782, SHARE-COVID19: GA N°101015924) and by DG Employment, Social Affairs & Inclusion through VS 2015/0195, VS 2016/0135, VS 2018/0285, VS 2019/0332, and VS 2020/0313. Additional funding from the German Ministry of Education and Research, the Max Planck Society for the Advancement of Science, the U.S. National Institute on Aging (U01_AG09740-13S2, P01_AG005842, P01_AG08291, P30_AG12815, R21_AG025169, Y1-AG-4553-01, IAG_BSR06-11, OGHA_04-064, HHSN271201300071C, RAG052527A) and from various national funding sources is gratefully acknowledged (see www.share-project.org).
