# Supplementary material for: Symptom Network Analysis Tools for Applied Researchers With Cross-Sectional and Panel Data – A Brief Overview and Multiverse Analysis
Source: Psychol Rep. 2023 Nov 9;128(6):4740–59. doi: 10.1177/00332941231213649 (PMC12480605; doi:10.1177/00332941231213649)
Supplement: Supplemental Material - Symptom Network Analysis Tools for Applied Researchers With Cross-Sectional and Panel Data – A Brief Overview and Multiverse Analysis [file sj-pdf-1-prx-10.1177_00332941231213649.pdf]

Symptom Network Analysis Tools for Applied Researchers with Cross-Sectional and Panel Data – A Brief Overview and Multiverse Analysis

Supplementary Materials

Supplementary Materials Section 1: Sample Characteristics 2

Supplementary Materials Section 2: Description of measures 3

Supplementary Materials Section 3: Model Stability Analysis 5

Supplementary Materials Section 1: Sample Characteristics

Sociodemographic characteristics for the sample used in all cross-sectional analyses are presented in Table S1.

Table S1

Sociodemographic sample characteristics at wave 1

Demographic information	Distribution
N	17945
% Female	55.05%
Age (Mean, SD)	63.66 (10.11)
Years of education (Mean, SD) *	10.59 (4.38)
Marital status (%)	
Married and living together with spouse	72.47
Registered partnership	1.76
Married, living separated from spouse	1.15
Never married	5.12
Divorced	6.04
Widowed	13.46

Note. Standard deviations are reported in parentheses. There was no valid information on years of education available for n = 5280.

The sample for the cross-lagged network model approach using complete case analysis consisted of 52344 individuals. We used full-information-maximum-likelihood estimation (FIML) with all available data for the estimation of the panel GVAR model.

Supplementary Materials Section 2: Description of measures

Depressive symptoms. The EURO-D scale (Prince et al., 1999) was used to assess depressive symptoms. The scale score is a composite sum score based on 12 items, including depression, pessimism, suicidality, guilt, sleep, interest, irritability, appetite, fatigue, concentration, enjoyment, and tearfulness (Maskileyson et al., 2021). All individual items evaluate symptom presence (0 = “not present”, 1 = “present”) in the past month. The sum score ranges from 0 (“not depressed”) to 12 (“very depressed”). The EURO-D scale has been shown to be a reliable and valid tool for detecting depression in older populations (Larraga et al., 2006).

Drinking frequency. The frequency of drinking alcohol beverages was assessed using a single-item measure. Respondents answered to the item “How often have you drunk any alcoholic beverages, like beer, cider, wine, spirits or cocktails?” on a 7-point scale (1 = *not at all,* 2: *less than once a month,* 3 = *once or twice a month,* 4 = *once or twice a week,* 5 = *three or four days a week,* 6 = *five or six days a week,* 7 = *almost every day*). The reference period was six-months in the first wave, and the last three months in all subsequent waves.

Quality of life. The CASP-12 measure (Wiggins et al., 2008) was used to assess quality of life. The CASP-12 total score summarizes four subscales (control, autonomy, pleasure, self-realization) and it ranges from 12 to 48. As a commonly used measure of quality of life in older populations, the CASP-12 has shown acceptable psychometric properties and prior studies supported the use of an overall sum score (Oliver et al., 2021).

Chronic diseases. A single item (“Has a doctor ever told you that you had ...”) was used to assess the presence and number of chronic diseases. The answer options included various chronic health conditions, including heart attack, high blood pressure or hypertension, high blood cholesterol, a stroke or vascular disease, diabetes, lung disease, cancer, Parkinson disease, different fractures (e.g., hip) and other conditions. Note that the answer options have

changed slightly across different waves, however, we have used a variable that only counts the conditions that were included in each wave.

Cognitive performance. Two tasks in the SHARE survey assessed cognitive performance.

Memory performance. Based on a word recall task (Harris & Dowson, 1982), two scores were extracted that describe the number of words recalled in the first trial, and the delayed condition of the task. The recall task assessed the immediate and delayed recall of a list of ten words. The two scores (immediate and delayed episodic memory) were averaged to create one aggregate score of memory performance. This score describes the proportion of words successfully recalled.

Mathematical performance. A percentage calculation task was used to assess participants' mathematical performance. The final numeracy test score is based on several items and describes the mathematical ability on a score ranging from 1 (poor) to 5 (good). Note that only participants present at baseline (wave 1) responded to the respective items.

Supplementary Materials Section 3: Model Stability Analysis

Figure S1

Results from non-parametric bootstrapping analysis (1000 bootstraps) for integrated cross-sectional network model

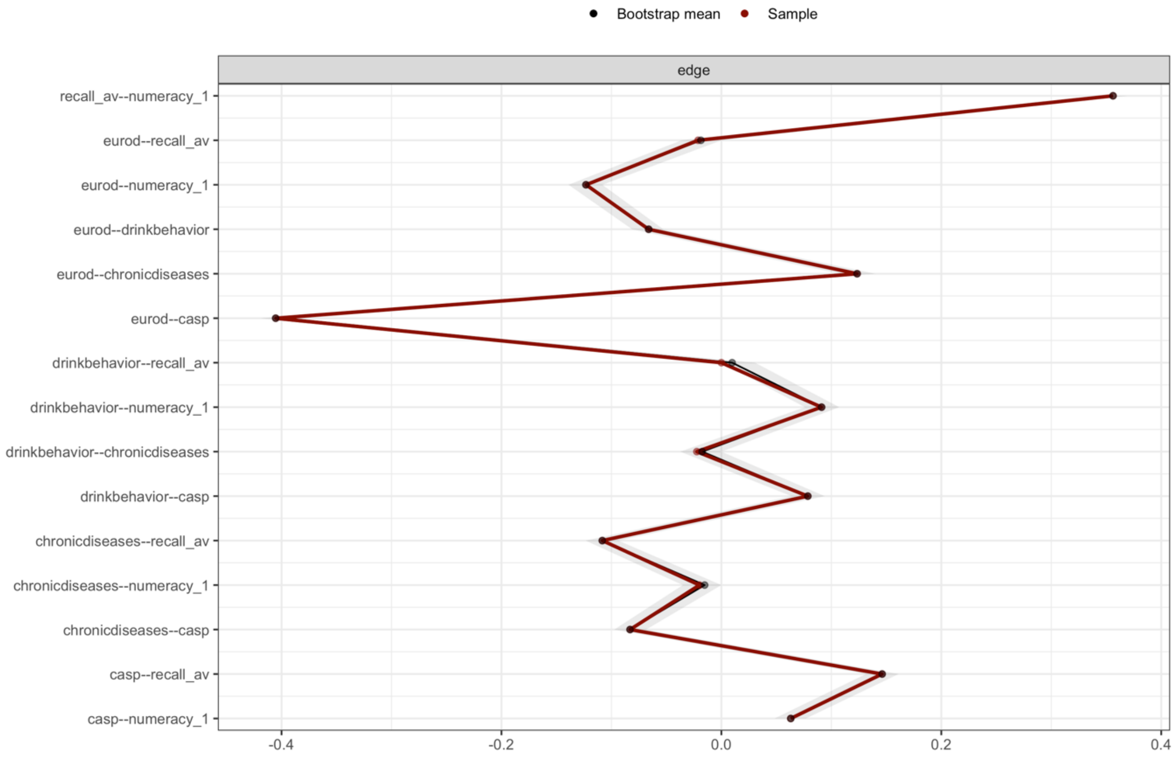

Note. recall_av = memory performance; numeracy_1 = mathematical performance; eurod = depressive symptoms; drinkbehavior = frequency of drinking; chronicdiseases = number of chronic diseases; casp = quality of life.

Figure S2

Bootstrapping results for moderated network model (1000 bootstraps)

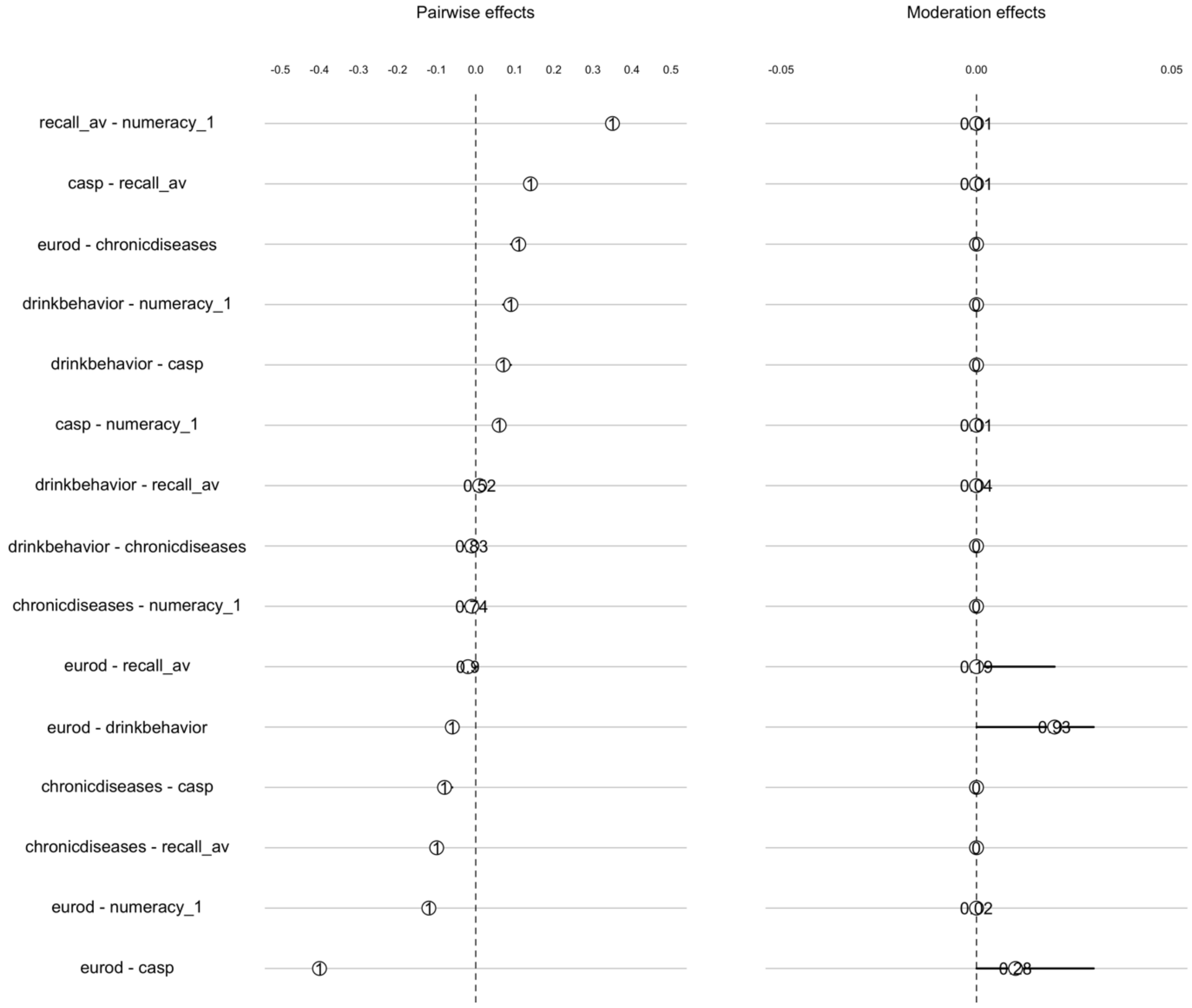

Note. The left panel displays refers to all pairwise associations, and the right panel refers to the moderation effect. Only the number of chronic diseases was included as a moderator. The horizontal interval indicates the variance of the estimates in the bootstrapped samples (5/95% quantiles shown). The proportion of non-zero parameters in the bootstrapped samples is shown in the figure. These results refer to our analysis using 1000 bootstrapped samples.

Figure S3

Bootstrapping results for panel GVAR temporal network

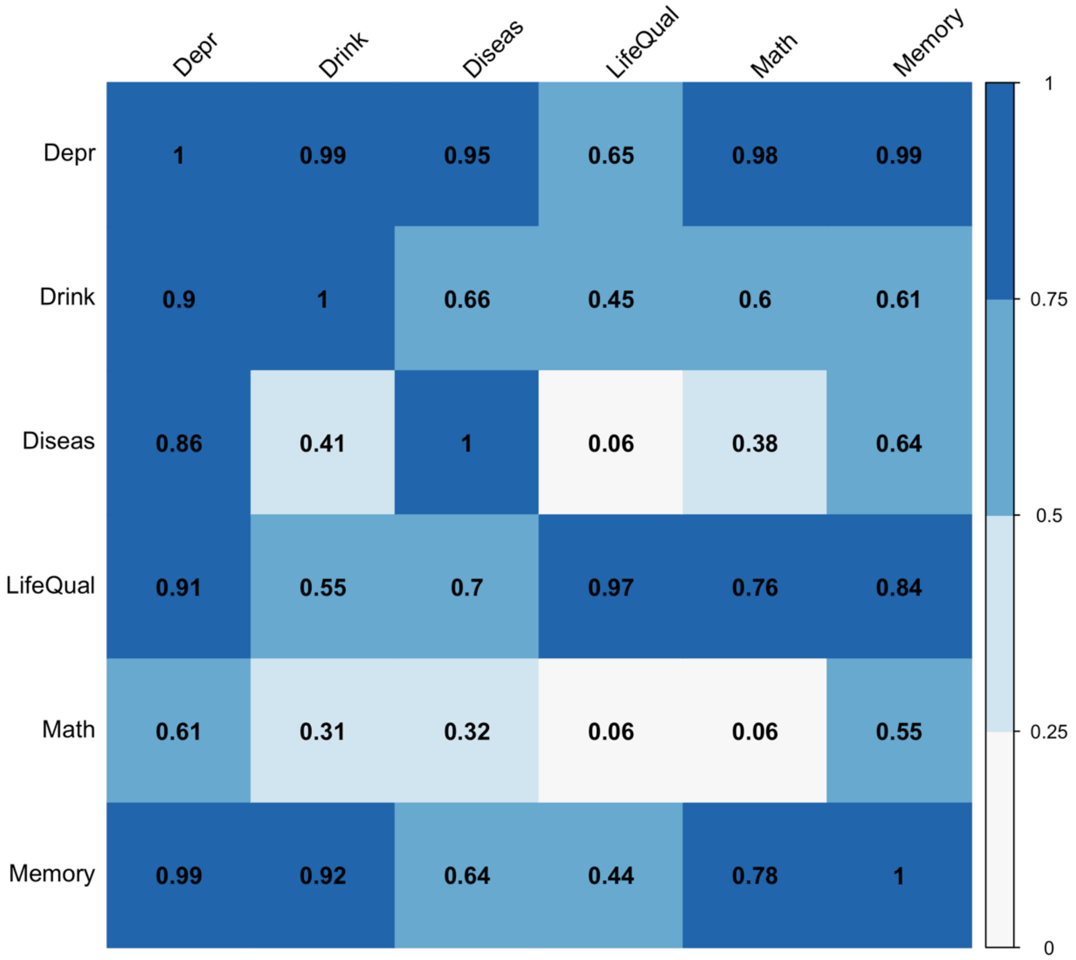

Note. The figure visualizes the proportion of times an edge is non-zero. The lower triangular of the figure refers to directed associations outgoing from the rows to the respective columns.

Figure S4

Bootstrapping results for panel GVAR contemporaneous network

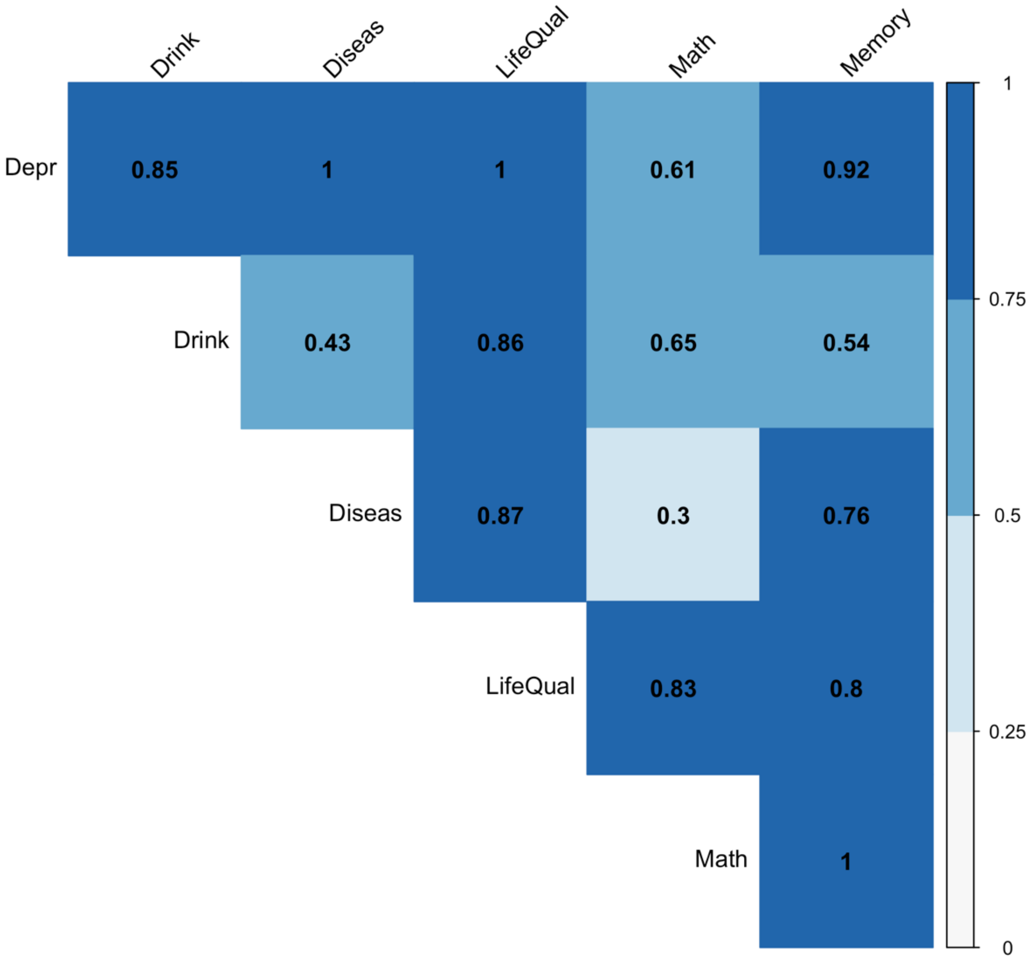

Note. The figure visualizes the proportion of times an edge is non-zero.

Figure S5

Bootstrapping results for panel GVAR between-person network

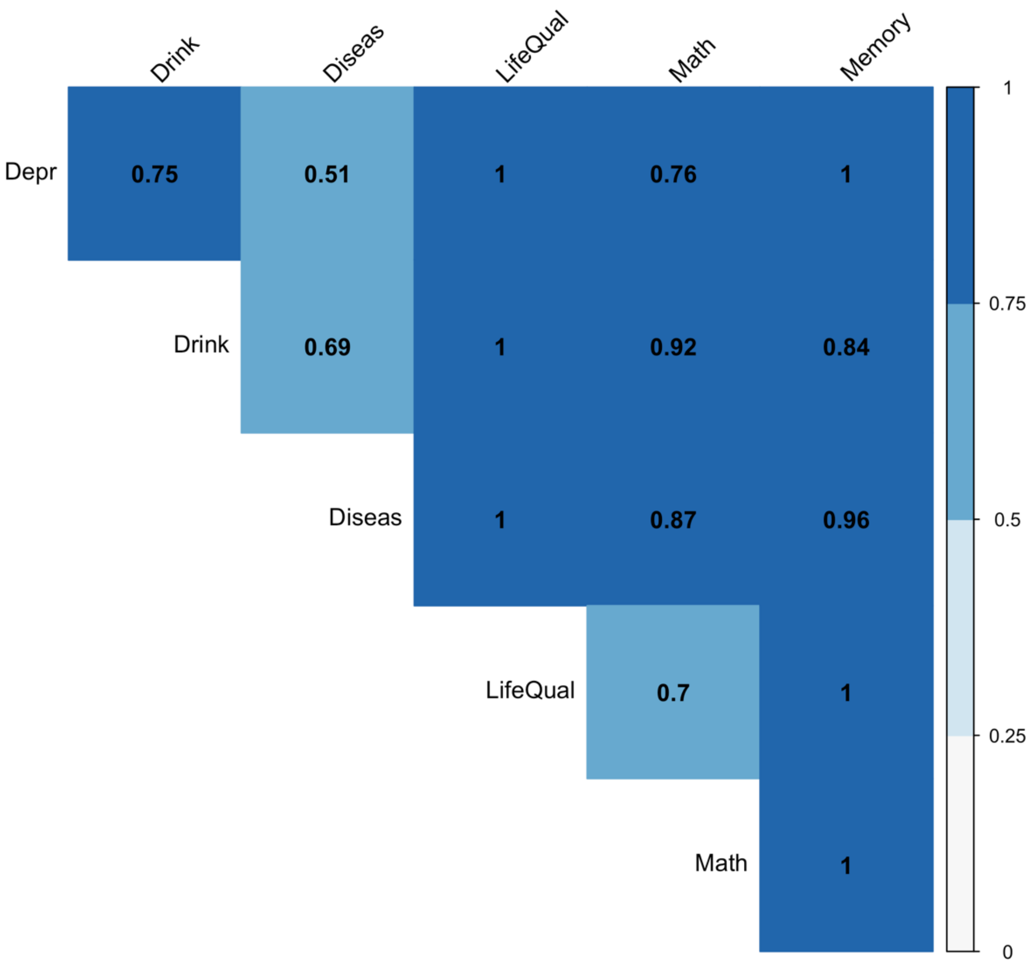

Note. The figure visualizes the proportion of times an edge is non-zero.

References

Harris, S. J., & Dowson, J. H. (1982). Recall of a 10-Word List in the Assessment of Dementia in the Elderly. *The British Journal of Psychiatry*, *141*(5), 524–527. https://doi.org/10.1192/bjp.141.5.524

Larraga, L., Saz, P., Dewey, M. E., Marcos, G., & Lobo, A. (2006). Validation of the Spanish version of the EURO-D scale: An instrument for detecting depression in older people. *International Journal of Geriatric Psychiatry*, *21*(12), 1199–1205. https://doi.org/10.1002/gps.1642

Maskileyson, D., Seddig, D., & Davidov, E. (2021). The EURO-D Measure of Depressive Symptoms in the Aging Population: Comparability Across European Countries and Israel. *Frontiers in Political Science*, *3*. https://www.frontiersin.org/articles/10.3389/fpos.2021.665004

Oliver, A., Sentandreu-Mañó, T., Tomás, J. M., Fernández, I., & Sancho, P. (2021). Quality of Life in European Older Adults of SHARE Wave 7: Comparing the Old and the Oldest-Old. *Journal of Clinical Medicine*, *10*(13), Article 13. https://doi.org/10.3390/jcm10132850

Prince, M. J., Beekman, A. T., Deeg, D. J., Fuhrer, R., Kivela, S. L., Lawlor, B. A., Lobo, A., Magnusson, H., Meller, I., van Oyen, H., Reischies, F., Roelands, M., Skoog, I., Turrina, C., & Copeland, J. R. (1999). Depression symptoms in late life assessed using the EURO-D scale. Effect of age, gender and marital status in 14 European centres. *The British Journal of Psychiatry: The Journal of Mental Science*, *174*, 339–345. https://doi.org/10.1192/bjp.174.4.339

Wiggins, R. D., Netuveli, G., Hyde, M., Higgs, P., & Blane, D. (2008). The Evaluation of a Self-enumerated Scale of Quality of Life (CASP-19) in the Context of Research on

Ageing: A Combination of Exploratory and Confirmatory Approaches. *Social Indicators Research*, *89*(1), 61–77. https://doi.org/10.1007/s11205-007-9220-5
